# Knowledge, Attitudes, and Practices towards Infectious Diseases Related to Travel of Community Pharmacists in Italy

**DOI:** 10.3390/ijerph17062147

**Published:** 2020-03-24

**Authors:** Giorgia Della Polla, Concetta Paola Pelullo, Francesco Napolitano, Chiara Lambiase, Caterina De Simone, Italo Francesco Angelillo

**Affiliations:** Department of Experimental Medicine, University of Campania “Luigi Vanvitelli”, Via L. Armanni, 5 80138 Naples, Italy; giorgia.dellapolla@unicampania.it (G.D.P.); concettapaola.pelullo@unicampania.it (C.P.P.); francesco.napolitano2@unicampania.it (F.N.); chiara.lambiase@gmail.com (C.L.); caterinadesimone14@gmail.com (C.D.S.)

**Keywords:** infectious diseases, Italy, pharmacists, practices, travel medicine

## Abstract

Pharmacists should be educated about travel medicine, since they could influence their own choices and those of the individuals they encounter. This study aims to investigate the knowledge, attitudes, and behaviors towards infectious diseases related to travel among community pharmacists in Italy. The data was collected from September 2018 to September 2019 using semi-structured telephone interviews. Only 1.8% answered correctly to all seven questions regarding the infectious diseases related to travel. Community pharmacists who had heard about travel medicine and those who had received information were more likely to have good knowledge. More than two-thirds of the respondents believed that it is important to provide information to the public about travel medicine. Pharmacists who worked a higher number of hours per week, were more knowledgeable about the more frequent infectious diseases related to travel, believed that travel medicine was a pharmacist competency, believed that they could give advice to the public, and had received information from scientific journals and educational activities were more likely to have this positive attitude. More than two-thirds often/always informed the public about the importance of having travel health center counseling. Pharmacists who had heard about travel medicine and those who believed that they could give advice to the public were more likely to inform. Interventions are needed to improve knowledge in order that community pharmacists can play an active role in counseling the public.

## 1. Introduction

During the past decade, the number of international travelers to many destinations in various geographic regions has steadily risen leading to an increased risk in travel-related health problems with a subsequent impact on international public health and an increased potential demand for health services [1,2]. Although the destination can be one important risk factor for certain health problems, many travelers are unaware and often fail to seek appropriate preventative pre-travel care.

Healthcare professionals have a central role in providing recommendations on travel diseases. In particular, primary care providers should be familiar with destination-specific disease risks, be knowledgeable in pre-travel health advice in order to reduce the risks for travelers, be prepared to prescribe and recommend medications for treatment that can be taken during the trip, and be able to identify those who might need additional follow-up after the trip [3]. Among healthcare professionals, pharmacists potentially have an important role in travel medicine within the community, and they are able to provide useful insights. In Italy, community pharmacists have a primary role also in providing recommendations to patients on the use of the healthcare services, and they can sell over-the-counter drugs to the public without medical counseling. Indeed, pharmacists can be one of the most common sources of healthcare information as they are closer to home and individuals often seek their services directly without consulting traveler medical centers. Therefore, pharmacists should be highly educated about travel medicine, since they could influence their own choices and those of the individuals they encounter. 

Several investigations on the knowledge, attitudes, and behavior of the general population and physicians regarding travel medicine have been conducted in different countries [4,5,6], and, to date, there is also research on the involvement of community pharmacists in this field [7,8,9,10,11,12,13,14,15,16]. This is of utmost importance in order to provide important information for planning training interventions targeted towards these professionals to positively affect travelers’ health. Therefore, to enhance the existing information, the current study investigated the level of knowledge, attitudes, and behaviors towards infectious diseases related to travel and their influencing factors among community pharmacists in Italy. 

## 2. Material and Methods

The study was one of the key components of a larger national survey and a detailed description of the procedure has been published previously [17] and is described in brief below.

### 2.1. Setting and Sample

This cross-sectional survey was conducted from September 2018 to September 2019, enrolling a random sample of 550 community pharmacists derived from the national list of those practicing in Italy. 

For the sample size calculation, the prevalence of respondents who believe that it is important to provide information to the public about infectious diseases related to travel was assumed to be 70%, with a significance level of 5%, a margin of error of 5%, and to account for a response rate of 70%. A sample size of 461 participants was calculated.

### 2.2. Procedure

Five trained and experienced interviewers initially contacted every selected community pharmacy by phone, and the pharmacist-in-charge or pharmacist manager were invited to participate in the survey. Data were collected from the individuals who responded. At the beginning of each telephone call, participants were informed about the purposes and confidentiality of the survey and data protection, that participation was voluntary, that they could not skip questions and whole sections, but they were free to terminate the call whenever they wished without any consequences. The selected person was able to indicate whether he/she wished to participate in the survey. All participants expressed their verbal informed consent to their inclusion before initiating the interview. To improve the response rate, additional telephone reminder calls with each of the pharmacists that were not able to complete the survey the first time were made every week. At least three additional calls were made at different times and days to reach a pharmacist before he/she was considered as a non-responder. Survey participation was not compensated. 

### 2.3. Survey Tool

Structured telephone interviews were designed to collect information divided into five sections. The first part consisted of the participants’ socio-demographic and professional characteristics and included questions regarding their age, gender, marital status, year of graduation, number of years in practice, and practice size. The second part consisted of questions related to the pharmacists’ knowledge about infectious diseases related to travel. Fourteen main infectious diseases selected according to the most frequently visited countries by the Italian population [5,18,19,20,21] were listed and pharmacists had to self-evaluate their knowledge giving their answers by choosing one of the three options: “Yes”, “No”, or “Don’t know”. The scale was then dichotomized (“Yes” = 1 and “No”/”Don’t know” = 0) and the total knowledge score for each participant was computed by adding up the scores (maximum score of 14). The total score was then categorized as poor knowledge (score of ≤2), moderate knowledge (score of 3–4), and good knowledge (score ≥5). The third part assessed participants’ attitudes with three statements on the importance of travel medicine for their work activity, asking whether they agreed that travel medicine is a pharmacist competency and whether they should give advice regarding travel medicine as part of their work activity, with response options on a 5-level Likert scale ranging from “Strongly disagree” to “Strongly agree”, and whether they believed that it is important to provide information to the public about travel medicine, with the response on a numerical 10-point Likert scale with higher values corresponding to a stronger attitude. The fourth part collected information about their practice and communication with the public regarding travel medicine. Three responses were collected on a 5-level Likert scale ranging from “Never” to “Always” and the others through multiple-choice alternatives. The fifth part included two questions on whether respondents had received information about travel medicine and whether they had educational needs. 

A pilot study for clarity, length, and simplicity was conducted on a total of 20 interviews and some minor wording changes were made in the revised version. These 20 pilot study interviews were not included in the main study. Then, the final protocol, including the informed consent form, and the questionnaire were approved by the Ethics Committee of the Teaching Hospital of the University of Campania “Luigi Vanvitelli”. 

### 2.4. Statistical Analysis

The statistical analysis was conducted using Stata statistical software, version 15 (StataCorp., College Station, TX, USA) [22]. First, descriptive analyses were performed to assess all characteristics of the participants. Second, chi-square and Student’s t-test were conducted to examine the relationship between the independent variables and the outcomes of interest. Third, multivariate ordered, linear, and logistic regression analysis was conducted by including in the models the variables with a *p*-value ≤ 0.25 at the bivariate analysis in order to estimate the independent association between potential predictors and the outcomes of interest. The following three models were developed: (1) pharmacists’ level of knowledge about the more frequent infectious diseases related to travel (poor = 1; moderate = 2; good = 3) (Model 1); (2) pharmacists who believed that it is important to provide information to the public about travel medicine (continuous) (Model 2); (3) pharmacists who often or always inform the public about the importance of having travel health center counseling (no = 0; yes = 1) (Model 3). Initial candidate variables included in all models were age (continuous), gender (male = 0; female = 1), marital status (unmarried/separated/divorced/widowed = 0; married = 1), number of years since degree (continuous), number of years in practice (continuous), number of hours worked per week (continuous), employment type (owner = 1; employee = 2; director = 3), having heard about travel medicine (no = 0; yes = 1), sources of information on travel medicine (none = 1; scientific journals and educational activities = 2; Internet and mass media = 3), and need of additional information on travel medicine (no = 0; yes = 1). Moreover, the following variables were also included: pharmacists’ level of knowledge about the more frequent infectious diseases related to travel (poor = 1; moderate = 2; good = 3), pharmacists who believed that travel medicine is a pharmacist competency (strongly disagree/disagree/uncertain = 0; agree/strongly agree = 1), pharmacists who believed that they could give advice to the public about travel medicine (strongly disagree/disagree/uncertain = 0; agree/strongly agree = 1) in Models 2 and 3, and pharmacists who believed that it is important to provide information to the public about travel medicine (continuous) in Model 3. A stepwise backward procedure was used to select which variables to include in the final models and in order to find the most parsimonious models. *P*-values of 0.2 and 0.4 were considered as a threshold to include and to eliminate variables. Odds ratios (ORs) and their respective 95% confidence intervals (CIs) were calculated using ordered and logistic regression. Standardized regression coefficients (β) were presented for the linear regression models. All tests were two-sided with *p*-values less than or equal to 0.05 considered statistically significant.

## 3. Results

### 3.1. Study Sample Characteristics

Among the 550 community pharmacists who were approached for the study, a total of 390 agreed to participate, resulting in a response rate of 70.9%. The socio-demographic and professional characteristics of the community pharmacists who participated in the survey are presented in Table 1. The majority of pharmacists were female (59.9%), the average age was 47.9 years, half of the sample were pharmacy owners (50.5%), the average number of years in practice was 18.1, and the mean number of hours worked per week was 41.2.

### 3.2. Knowledge

Regarding the level of knowledge, an overwhelming majority of respondents were aware of travel medicine (85.4%). Notably, the highest achieved score on the knowledge of infectious diseases related to travel was 7 out of a maximum score of 14, and only 1.8% provided correct answers to seven questions and were aware of none of these diseases. Travel diarrhea (91.9%), hepatitis A (44.5%), malaria (44.3%), and cholera (36.5%) were the more recurrent diseases known by pharmacists. For all participants, the total score for knowledge of infectious diseases related to travel ranged between 0 and 7, with a mean of 2.9. Associations between the different outcomes of interest and potential predictor variables using multivariable linear and logistic regression analyses are shown in Table 2. From the initial model and after the stepwise backward procedure, the final ordered logistic regression model with the outcome the score of the knowledge about the infectious diseases related to travel comprised two variables: level of knowledge and sources of information about travel medicine. Pharmacists who had heard about travel medicine (OR = 2.28; 95% CI = 1.26–4.11), having received information from scientific journals and educational activities (OR = 5.57; 95% CI = 3.42–9.1) and from the Internet and mass media (OR = 3.96; 95% CI = 2.37–6.6), compared with those who did not receive information, were more likely to have a good knowledge about the more frequent infectious diseases related to travel (Model 1).

### 3.3. Attitudes

With regard to attitudes, 44.3% and 56.2% of the respondents agreed respectively with the statements that travel medicine is part of their professional responsibilities as healthcare workers and that they could properly give advice to the public on this topic. Moreover, more than two-thirds of respondents (77.6%) believed that it is important to provide information to the public about travel medicine, with a mean value of 6.9, on a scale of 1 to 10. The results of the multivariate linear regression model, built to test the variables associated with this outcome of interest, showed that pharmacists who worked a higher number of hours for week, those who had a higher level of knowledge about the more frequent infectious diseases related to travel, those who believed that travel medicine is a pharmacists’ competence, those who believed that they could give advice to the public about travel medicine, and those who had received information from scientific journals and educational activities compared with those who did not receive any information were more likely to believe that it is important to provide information to the public about travel medicine (Model 2 in Table 2).

### 3.4. Behaviors

Regarding the behaviors, more than one-third of the sample (37.2%) indicated that they sometimes receive requests for advice on travel medicine from the public, mainly regarding travel diarrhea (83.4%), safeness of food and water (77.6%), insect punctures (65.9%), and vaccinations (31.2%). Moreover, more than half (52.1%) reported that the public often ask for medications for travel purposes without a prescription. The reasons for these requests included the inability to get a prescription in time (76.5%), advised information from the Internet (47%), and unavailability of the physician (19.1%). More than two-thirds (69.5%) often or always informed the public about the importance of having travel health center counseling. Multivariate logistic regression analysis showed that pharmacists who had heard about travel medicine (OR = 6.44; 95% CI 3.2–12.97) and those who believed that they could appropriately give advice to the public about travel medicine (OR = 3.61; 95% CI 1.9–6.84) were more likely to inform the public about the importance of having travel health center counseling (Model 3 in Table 2).

### 3.5. Sources of Information

Among the survey participants, only two-thirds (65.6%) reported searching for information about travel medicine. When asked about the source of information, a higher number of respondents stated that the Internet was the most frequently reported trusted source (52.9%), followed by scientific journals (27.9%), and educational activities (20.7%). The vast majority of responders (84.6%) felt they did not have sufficient information on travel medicine and expressed willingness to acquire more knowledge.

## 4. Discussion

The present study is to our knowledge the first and largest published national survey that has attempted to provide an overall picture about the knowledge, attitudes, and practices of community pharmacists in Italy regarding infectious diseases related to travel and of the factors associated with these main outcomes of interest, adding to the existing literature by providing key findings.

The study findings suggest that the participants had a knowledge gap in travel medicine and this situation is alarming as a basic level of knowledge about common infectious diseases is expected from healthcare staff. Indeed, a striking finding depicted that less than a quarter of participants seemed knowledgeable about the seven more frequent infectious diseases related to travel, with a total mean score of knowledge of 2.9. The finding that respondents had a lack of knowledge is in accordance with previous reports, which have shown that community pharmacists have a knowledge gap and low confidence in providing this type of care [15,23]. This fact emphasizes the importance that pharmacists should be informed since their level of awareness should primarily be improved. Moreover, pharmacists who had a higher level of knowledge were far more likely to believe that it is important to provide information to the public about travel medicine and to inform them about the potential risks of travel diseases and the importance of having travel health center counseling before their departure. Therefore, it is necessary that all pharmacists choose to improve their knowledge in order to offer travelers accurate and expert counselling.

With respect to the attitudes towards travel medicine, this study indicated that 44.3% and 56.2% of the respondents agreed that travel medicine is part of their professional responsibilities as healthcare workers and that they could properly give advice to the public. A similar result was observed among Malaysian community pharmacists who agreed that they are in a position to provide health information and recommendations regarding travel health [7]. This is a relevant finding because pharmacists should give advice to patients in order to guarantee the appropriate behaviors before and during their travels and they may be a key and trusted source of information. Multivariate linear regression analysis showed that pharmacists who believed that knowledge about travel medicine is a part of their role were more likely to believe that it is important that they provide information to the public. This result can be explained by the fact that pharmacists who had this attitude were more likely to acquire information because they thought travel medicine was a key professional skill. Therefore, policy makers and healthcare managers should implement programs that actively involve the pharmacists because pharmaceutical services are available throughout the territory and can easily be accessed, and this could lead to a greater adherence of the public to preventive measures and may contribute to patient safety and appropriate use of medications.

Concerning the practices for travelers, community pharmacists play a key role in the public’s awareness of travel diseases and their recommendations may be an important determinant of travel health. In this study, 69.5% of the participants often or always informed the public about the importance of having a travel health center counselling session. This underlined the availability of the community pharmacists in providing information to the public regarding travel diseases. However, it is important to highlight that in Italy pharmacists cannot dispense some medicines without a physician’s prescription, and it should be considered that more than half reported that the public often asked for medications for imminent travel because their general practitioners were unavailable. This result is in line with those of a similar study conducted in Australia that showed that pharmacists had a role in advising travelers, who would not normally visit a physician before travelling, on travel-related health issues before visiting certain destinations [24]. Furthermore, the result of the logistic regression analysis showed that the positive attitude of the study participants about different health seeking behaviors predicted their real practices since those who believed that they could give advice to the public regarding travel medicine were more likely to often or always inform the public about the importance of having travel health center counselling than those who did not. 

The findings from this survey are consistent with several previous results from similar studies among different groups of individuals that have demonstrated the importance of scientific information for their knowledge and attitudes. Indeed, as described in prior studies [5,17,25,26,27,28,29], it is evident from the multivariate analysis that receiving information from scientific journals and educational activities facilitates the emergence of pharmacists who have better knowledge and have more positive attitudes. This is important because the pharmacists themselves, in order to effectively influence others, must be well equipped with an appropriate level of knowledge, and interventions should be aimed at including such sources as an important conduit of travel medicine-related information, which can support the pharmacists’ confidence. However, an unsurprising finding was that a large majority of pharmacists identified the Internet as being their main source of information, rather than the medical establishment. It should be underlined that several studies have expressed concern about the quality and accuracy of health information on the web [30,31,32], and, therefore, it may not provide all of the details necessary to allow the pharmacists to make well-informed suggestions. Consequently, it is possible that there are missed opportunities for pharmacists to provide high quality information. Health information via the Internet should not replace other healthcare professional experts in travel medicine who can give advice for specific situations. Furthermore, the vast majority of responders felt they did not have sufficient information to adequately answer questions on this field. Clear communication and knowledge about travel medicine would help to instill confidence in pharmacists and keep them adequately informed in order to meet the needs of their community, as well as enable them to discuss any concerns with their customers that may arise from what their customers have read. Therefore, pharmacists must have the capacity needed to effectively educate and address public questions and concerns since the delivery of information from a trusted or known source with which the individual has already developed a relationship may be beneficial. This is crucial also in light of the measures taken for the Coronavirus disease (COVID-19) by the Italian authorities [33], as pharmacists represent an important element in order to provide appropriate recommendations to the public about preventive measures. 

### Limitations

When considering the study findings, it is important to take into account some potential methodological limitations such as those normally observed in similar survey-based studies. First, the cross-sectional design of this study can only demonstrate associations between the different outcomes of interest and the observed determinants, and it is not possible to say anything about causality. Second, data collection through the telephone survey was based on self-reported information, and so may be subject to reporting bias. Third, there is also the risk for social desirability bias, by which participants do not report attitudes and behaviors fully or accurately and may provide the responses they believe the researcher wants to know instead of the truth, as opposed to answering honestly, and it is possible that they may over-report socially desirable attitudes and behaviors or under-report socially undesirable attitudes and behaviors. Efforts were made to minimize the risk of these biases by ensuring participants that the study was anonymous and confidential and that their data would be de-identified. Fourth, it is possible that pharmacists with specific positive or negative opinions or interest in the topic were more or less likely to respond to the survey. If such bias exists, it may lead to an over- or underrepresentation of the rate of positive responses. However, the high response rate could offset this bias. Moreover, no difference has been observed between respondents and non-respondents regarding the geographic area of activity. 

## 5. Conclusions 

In conclusion, this survey provides some insights into the knowledge, attitudes, and behaviors regarding infectious diseases related to travel among community pharmacists in Italy and identifies their associated characteristics. The findings will prove useful when designing and implementing targeted interventions to improve the level of knowledge of pharmacists so that they can play an active role in counseling the public and also in working more closely with the health services in travel medicine. 

## Figures and Tables

**Table 1:** Socio-demographic and professional characteristics of the study population.

Characteristics	N	%
Age, years	47.9 ± 11.1 (25–79) *	
Gender		
Male	155	40.1
Female	232	59.9
Marital status		
Married	213	54.6
Unmarried/separated/divorced/widowed	177	45.4
Number of years since degree	21.2 ± 11.1 (1–54) *	
Number of years in practice	18.1 ± 11.2 (1–45) *	
Number of hours worked per week	41.2 ± 9.3 (1–72) *	
Role in the pharmacy		
Owner	196	50.5
Employee	146	37.6
Director	46	11.9

Number for each item may not add up to total number of study population due to missing values; * Mean ± standard deviation (range).

**Table 2:** Multivariate ordered logistic, linear, and logistic regression analyses to characterize factors associated with the different outcome of interest.

**Variable**	**OR**	**SE**	**95% CI**	***p***
**Model 1. Pharmacists who had a good knowledge about the more frequent infectious diseases related to travel**				
Log likelihood = −366.9, χ^2^ = 71.15 (7 df), *p* < 0.0001				
Sources of information				
None	1 *			
Scientific journals and educational activities	5.57	1.39	3.42–9.1	<0.001
Internet and mass media	3.96	1.03	2.37–6.6	<0.001
Pharmacists who had heard about travel medicine	2.28	0.69	1.26–4.11	0.006
Pharmacists who had higher number of years in practice	1.02	0.01	0.99–1.05	0.184
Pharmacists who felt the need for additional information about travel medicine	1.39	0.4	0.79–2.44	0.245
Pharmacists who had lower number of years since degree	0.99	0.02	0.94–1.04	0.756
Older age	1.01	0.02	0.95–1.06	0.84
**Variable**	**COEFF.**	**SE**	**t**	***p***
**Model 2. Pharmacists who believed that it is important to provide information to the public about travel medicine**				
F = 27.95, R^2^ = 3.1%, adjusted R^2^ = 3%, *p* < 0.0001				
Pharmacists’ level of knowledge about the more frequent infectious diseases related to travel	0.86	0.18	4.64	<0.001
Poor	1 *			
Good	0.86	0.18	4.64	<0.001
Pharmacists who believed that travel medicine is a pharmacist competency	1.25	0.17	7.14	<0.001
Pharmacists who believed that they could give advice to the public about travel medicine	0.61	0.18	3.43	0.001
Sources of information				
None	1 *			
Scientific journals and educational activities	0.4	0.16	2.57	0.01
Pharmacists who worked a higher number of hours for week	0.02	0.008	2.25	0.025
Male	0.23	0.15	1.55	0.121
**Variable**	**OR**	**SE**	**95% CI**	***p***
**Model 3. Pharmacists who often or always informed the public about the importance of having travel health center counseling**				
Log likelihood = −191.74, χ^2^ = 76.64 (7 df), *p* < 0.0001				
Pharmacists who had heard about travel medicine	6.44	2.3	3.2–12.97	<0.001
Pharmacists who believed that they could give advice to the public about travel medicine	3.61	1.18	1.9–6.84	<0.001
Pharmacists who believed that it is important to provide information to the public about travel medicine	1.12	0.9	0.94–1.33	0.191
Pharmacists who did not believe that travel medicine is a pharmacist competency	0.65	0.22	0.33–1.28	0.217
Female	1.33	0.34	0.81–2.21	0.261
Pharmacists who felt the need for additional information about travel medicine	1.46	0.55	0.7–3.06	0.308
Pharmacists who had higher number of years in practice	1.01	0.01	0.99–1.03	0.372

* Reference category. OR: Odds Ratio; SE: Standard Error; COEFF: Coefficient.
